# When Blood in the Belly Stops the Bowel: Hemorrhagic Ascites Causing Ileus

**DOI:** 10.7759/cureus.109392

**Published:** 2026-05-21

**Authors:** E. Casey Anders, Pooja Parikh, James Barefield, Amet Diedhiou

**Affiliations:** 1 Internal Medicine, Prisma Health Midlands, Columbia, USA; 2 Internal Medicine, University of Southern California School of Medicine, Columbia, USA

**Keywords:** ascites, ascites-induced ileus, hemorrhagic ascites, ileus, pancreatic ductal leak

## Abstract

Ascites is a common cause of abdominal distension and may contribute to increased intra-abdominal pressure and impaired gastrointestinal motility. Although ileus is more commonly associated with postoperative states, infection, medications, or electrolyte disturbances, massive ascites can rarely result in a functional bowel obstruction through elevated intra-abdominal pressure. We present a patient with a low serum ascites albumin gradient (SAAG) secondary to a pancreatic ductal leak in the setting of chronic pancreatitis who developed progressive abdominal distension, nausea, and obstipation. Imaging demonstrated diffuse bowel dilation without a clear transition point or evidence of mechanical obstruction. In this case, pancreatic ascites was the primary driver of intra-abdominal hypertension. Following large-volume paracentesis and endoscopic retrograde cholangiopancreatography (ERCP) intervention, the patient experienced rapid symptomatic improvement and return of bowel function, supporting a diagnosis of ascites-induced ileus secondary to intra-abdominal hypertension. This case highlights an uncommon complication of pancreatic ascites and underscores the importance of recognizing elevated intra-abdominal pressure as a reversible cause of ileus.

## Introduction

Ascites, defined as the pathological accumulation of fluid within the peritoneal cavity, most commonly results from portal hypertension in the setting of advanced liver disease, but may also arise from malignancy, infection, or lymphatic obstruction [1]. A less common but clinically important etiology is pancreatic ascites, which results from the leakage of pancreatic secretions into the peritoneal cavity due to the disruption of the pancreatic duct or the rupture of a pancreatic pseudocyst [2]. It should be suspected in patients with a history of chronic pancreatitis, alcohol use disorder, or abdominal trauma who present with recurrent, refractory, or unexplained ascites [3]. Typical symptoms include abdominal pain, early satiety, abdominal distention, weight loss, and recurrent ascites [2]. 

As ascitic volume increases, intra-abdominal pressure may rise, predisposing patients to intra-abdominal hypertension and, in severe cases, abdominal compartment syndrome [1]. Intra-abdominal hypertension is defined as a sustained intra-abdominal pressure >12 mmHg, while abdominal compartment syndrome is defined as intra-abdominal pressure >20 mmHg with associated organ dysfunction [4]. It has been demonstrated that excessive ascitic fluid can exert extrinsic compression on the intestines, leading to decreased mesenteric perfusion, intestinal wall edema, and hypomotility [4]. Clinically, this may present with abdominal distension, nausea, and obstipation consistent with ileus. Therapeutic paracentesis often yields rapid symptom resolution, supporting the causal role of elevated intra-abdominal pressure in ileus development.

We report a case of massive hemorrhagic ascites secondary to pancreatic ductal disruption in which functional ileus dominated the clinical presentation. Decompressive paracentesis led to rapid improvement in bowel function. The pathophysiological mechanisms, diagnostic challenges, and management considerations of this uncommon presentation are discussed.

## Case presentation

A 38-year-old man with a history of polysubstance use disorder and alcohol-induced pancreatitis status post embolization for pancreatic hemorrhage presented to a rural emergency department (ED) with diffuse abdominal and flank pain, nausea, and vomiting. The pain was described as an aching, burning, and pressure-like sensation in the epigastric region radiating to the back. He had undergone a therapeutic paracentesis the previous day, during which 8.4 liters of ascitic fluid were removed. 

On arrival to the ED, he had a temperature of 97.7°F, a heart rate of 105 bpm, a respiratory rate of 12 breaths/min, a blood pressure of 119/97 mmHg, and an oxygen saturation of 98% on room air. Laboratory studies revealed leukocytosis (white blood cell (WBC) 11×10³/µL), hyponatremia (Na 127 mmol/L), low serum bicarbonate (20 mmol/L), and an elevated lipase (177 U/L). Computed tomography (CT) of the abdomen and pelvis demonstrated chronic pancreatic changes with cystic lesions in the uncinate process and tail, moderate ascites with possible loculated pockets, and mild proximal colonic dilatation consistent with ileus. He was started empirically on cefepime and metronidazole for presumed spontaneous bacterial peritonitis (SBP), and a nasogastric (NG) tube was placed to low-intermittent suction. Diagnostic paracentesis was performed, and he was admitted for further management. 

Over subsequent days, he received IV fluids, antibiotics, and spironolactone 50 mg twice daily. Pain was managed with IV hydromorphone. Peritoneal fluid analysis revealed an amber colored fluid, red blood cell (RBC) 11,000 µL, total nucleated cell (TNC) 559 µL, and amylase 937 U/L. There was no evidence of SBP, so antibiotics were discontinued. However, the presence of significant RBCs raised concern for hemorrhagic ascites. 

**Figure 1:**
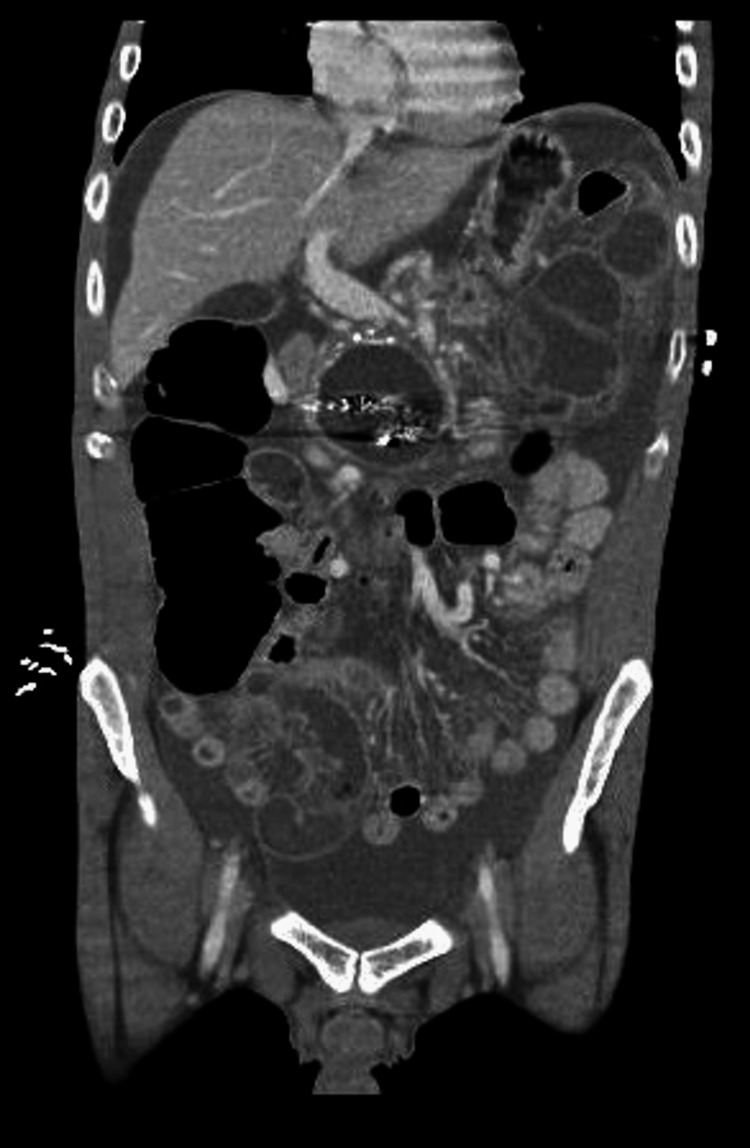
Computed tomography showing mild dilation of the proximal colon, concerning for ileus

Repeat CT showed increased ascites, a new left pleural effusion, and multiple cystic lesions near the pancreatic head consistent with sequelae of chronic pancreatitis versus cystic neoplasm. Given the complexity of findings, general surgery recommended transfer to a tertiary center (Figure 1).

At the tertiary facility, total parenteral nutrition (TPN) was initiated, and repeat paracentesis removed 5 liters of fluid. Computed tomography angiography (CTA) ruled out pseudoaneurysm but confirmed multiple pancreatic pseudocysts and mild ascites. Endoscopic retrograde cholangiopancreatography (ERCP) demonstrated contrast extravasation from the pancreatic duct in the tail, confirming a pancreatic ductal leak. Attempts at guidewire passage were unsuccessful, and pancreatic and biliary sphincterotomies were performed. Endoscopic ultrasound (EUS) confirmed chronic pancreatitis with pancreatic duct dilation (5 mm).

These findings established the diagnosis of pancreatic duct disruption with persistent pancreatic ascites.

During hospitalization, he developed supraventricular tachycardia (SVT), requiring adenosine and diltiazem. This was attributed to metabolic stress from pancreatitis in the setting of metabolic derangements. Over time, his ascites and pseudocysts increased, and additional paracenteses (3.5 L and 4.5L) were performed. Peritoneal fluid analysis showed yellow fluid with RBC 271 µL, TNC 31 µL, albumin 1 g/dL, amylase 56 U/L, and protein 2.6 g/dL. Serum amylase was 229 U/L. The serum ascites albumin gradient (SAAG) was <1.1 g/dL, consistent with a non-portal hypertensive etiology.

Blood cultures grew *Escherichia coli*, presumed bacterial translocation from complicated pancreatitis. The *E. coli *was pan-sensitive, so he was treated with ceftriaxone and metronidazole. Later, EUS-guided cystogastrostomy stent placement was performed to drain walled-off necrosis.

Right upper quadrant ultrasound revealed cirrhotic liver morphology with patent vasculature and large-volume ascites. However, the SAAG was not consistent with portal hypertension. The patient improved clinically and was discharged with a peripherally inserted central catheter (PICC) line on ciprofloxacin, ceftriaxone, and metronidazole for a 14-day course, with close outpatient follow-up.

Table 1 presents the patient's timeline of hospitalization.

**Table 1:** Timeline of hospitalization highlighting when paracentesis was completed and imaging findings CT: computed tomography; IR: interventional radiology; RBC: red blood cell; TNC: total nucleated cell; SBO: small bowel obstruction; TPN: total parenteral nutrition; ERCP: endoscopic retrograde cholangiopancreatography; EUS: endoscopic ultrasound; SAAG: serum ascites albumin gradient

Day	Paracentesis/imaging performed
1 day prior to admission	Large-volume paracentesis, 8.4 L removed
Hospital day 1	CT of the abdomen concerning for ileus
Hospital day 2	IR-guided paracentesis, 1.6 L removed. Ascitic fluid analysis: amber-colored fluid, RBC 11,000 µL, TNC 559 µL, and amylase 937 U/L
Hospital day 3	Abdominal X-ray showed mild colonic ileus
Hospital day 6	IR-guided paracentesis, 3.2 L removed
Hospital day 7	Surgery was consulted for ileus vs. SBO and determined ileus secondary to complicated pancreatitis and hemorrhagic ascites
Hospital day 8	TPN initiated
Hospital day 9	IR-guided paracentesis, 3.8 L removed. CT angiogram showed stable cystic pancreatic lesions (tail and uncinate region, 6.5 cm uncinate lesion with metallic foci from prior coil embolization), mild ascites, and no active vascular aneurysm. Transferred to a tertiary center
Hospital day 10	ERCP was performed and showed contrast extravasation from the pancreatic duct in the tail consistent with ductal leak. Sphincterotomies were performed at the biliary and pancreatic orifices. Diagnosis of pancreatic ductal leak was confirmed. EUS was performed and showed chronic pancreatitis changes in the pancreatic head and pancreatic ductal dilation
Hospital day 13	Bedside paracentesis, 3.5 L removed. Ascitic fluid analysis: yellow-colored fluid, RBC 271 µL, TNC 31 µL, albumin 1 g/dL, protein 2.6 g/dL, and amylase 56 U/L. Serum amylase was 229 U/L. SAAG was <1
Hospital day 14	Abdominal X-ray showed improving colonic gaseous distention
Hospital day 16	Abdominal X-ray showed persistent but slightly improved transverse colon dilation
Hospital day 19	CT of the abdomen and pelvis showed no evidence of ileus
Hospital day 21	EUS was performed: cystogastrostomy stent was placed
Hospital day 22	CT-guided paracentesis performed, 4.3 L removed. Abdominal ultrasound showed heterogeneous liver echotexture with nodular contour
Hospital day 31	IR-guided paracentesis, 3.2 L removed

## Discussion

This case illustrates a rare but clinically significant complication of chronic alcohol-related pancreatitis, hemorrhagic pancreatic ascites secondary to pancreatic duct disruption, complicated by functional ileus due to intra-abdominal hypertension physiology.

Pancreatic ascites results from the disruption of the pancreatic duct, allowing enzyme-rich fluid to leak into the peritoneal cavity. Though uncommon, occurring in approximately 1% of patients with chronic pancreatic cases, it is more frequently observed in males with long-standing alcohol use disorder and may also occur following trauma or acute necrotizing pancreatitis [2]. Diagnosis is established by ascitic fluid analysis demonstrating high amylase (often >3× serum levels), high protein content (often >3 g/dL), and low SAAG (<1.1 g/dL) [2,3,5]. Hemorrhagic ascites, RBCs >10,000 µL in the ascitic fluid, may result from pseudocyst erosion or peritoneal bleeding, distinguishing it from cirrhotic or malignant etiologies. Hemorrhagic ascites can be distinguished from a traumatic tap by its heterogeneous, non-clotting nature.

Although ultrasound suggested cirrhotic morphology, the consistently low SAAG and markedly elevated ascitic amylase supported a pancreatic, non-portal hypertensive etiology.

Definitive management of pancreatic ascites focuses on reducing pancreatic ductal pressure and controlling the source of leakage. Conservative therapy includes bowel rest, TPN, octreotide, and serial paracenteses [3]. While approximately one-third of patients may respond to conservative management, endoscopic interventions such as ERCP with transpapillary stenting, sphincterotomy, or EUS-guided drainage are increasingly first-line for persistent leaks [2,6]. ERCP not only confirms the diagnosis by directly visualizing pancreatic duct disruption but also provides therapeutic benefit through ductal decompression with either sphincterotomy or stent placement, thereby reducing intra-abdominal pressure and ongoing fluid accumulation [5].

The ileus observed in this case was likely multifactorial. Massive ascites contributed to increased intra-abdominal pressure with resultant bowel wall edema and impaired motility. This was compounded by opioid exposure, which further reduced gastrointestinal peristalsis, and systemic infection from *E. coli *bacteremia, which may have contributed to inflammation-mediated gut dysmotility.

## Conclusions

This case highlights the diagnostic and therapeutic challenges of hemorrhagic pancreatic ascites complicating chronic pancreatitis. Clinicians should maintain a high index of suspicion for pancreatic duct disruption in patients with recurrent or hemorrhagic ascites, particularly in the setting of low SAAG and elevated ascitic amylase. 

Early recognition with comprehensive ascitic fluid analysis and advanced imaging is essential for diagnosis. Multidisciplinary management and timely endoscopy intervention, including ERCP with ductal stenting and EUS-guided drainage, are critical in reducing morbidity and preventing recurrence. Importantly, this case emphasizes that massive ascites may precipitate functional ileus through intra-abdominal hypertension physiology and that therapeutic paracentesis can provide both diagnostic clarity and rapid symptomatic improvement. 
